# Dynamic obstacle detection method based on U–V disparity and residual optical flow for autonomous driving

**DOI:** 10.1038/s41598-023-34777-6

**Published:** 2023-05-10

**Authors:** Jianying Yuan, Tao Jiang, Xi He, Sidong Wu, Jiajia Liu, Dequan Guo

**Affiliations:** 1 Chengdu University of Information Technology, Chengdu, China; 2 Chengdu University of Traditional Chinese Medicine, Chengdu, China

**Keywords:** Engineering, Mathematics and computing

## Abstract

As a core step of obstacle avoidance and path planning, dynamic obstacle detection is critical for autonomous driving. This study aimed to propose a dynamic obstacle detection method based on U–V disparity and residual optical flow for autonomous driving. First, a drivable area of an unmanned vehicle was detected using U–V disparity images. Then, obstacles in the drivable area were detected using U–V disparity images and the geometric relationship between obstacle size and its disparity. Finally, the motion likelihood of each obstacle was estimated by compensating the camera ego-motion. The innovation of the proposed method was that the searching range of the moving obstacles was greatly narrowed by detecting the obstacles in the drivable area, which greatly improved not only the moving obstacle detection efficiency but also the detection accuracy. Datasets from the KITTI benchmark and our self-acquired campus scene data were chosen as testing samples. The experimental results showed that our method could achieve high detection precision, low missed detection rate and less time consumption.

## Introduction

Autonomous driving has been an active field of intelligent transportation systems in the last decade^1^. The core technologies of autonomous driving include environmental perception, navigation and localization, path planning, and decision-making. The moving object detection is one of the key parts of environmental perception because compared with static traffic facilities, moving objects in traffic scenes pose more threats to driving safety moving object detection method based on LiDAR, vision-based moving object detection method has a lower cost and is closer to human perception. For automatic driving moving object detection methods, it can be divided into traditional methods and deep learning-based method. Although deep learning-based methods have strong adaptability to different traffic environments, they require a large number of training data and the calculation process is opaque. Therefore, this paper focuses on building a moving object detection system for autonomous driving using traditional computer vision algorithms.

In traditional moving objects detection method, both monocular-based^1–6^ and binocular cameras-based^7–18^ method were studied. The former uses only one camera and needs less computation, but only one constraint is not suitable for all scenarios because it can easily fail in degeneration^7^. Besides, the scale information cannot be recovered. The latter uses stereo cameras, and gets the moving states by extracting the residual optical flow (ROF) between two consecutive images. Both sparse and dense optical flow vectors can be used to extract the ROF^19^. However, one strategy the two methods have in common is that they search for moving obstacles in the entire image. This strategy has two disadvantages. For an unmanned vehicle, it usually focuses only on the moving obstacles on the road in front of it. Therefore, if the searching area is in the whole image, it increases the search range and the system time cost on the one hand and increases the false detection rate of nonmoving obstacles and reduces the detection accuracy of the system on the other hand. Therefore, a dynamic obstacle detection method based on U–V disparity and ROF was proposed in this study to improve the detection accuracy and efficiency of the existing moving object detection algorithms. The core idea of the proposed algorithm was to combine the detection of the drivable area of the unmanned vehicle, the detection of obstacles, and the estimation of the ROF. First, the obstacles on the road ahead of the unmanned vehicle were detected using the U–V disparity, and then the motion state of each obstacle was determined using their ROF. The main contributions of this study were as follows:

1. A highly accurate and efficient method for dynamic obstacle detection was proposed. The motion state of the obstacle in the passable area could be calculated directly by calculating the U–V disparity and ROF of two adjacent stereo images. Compared with the traditional method based on only the ROF, the proposed method not only narrowed the dynamic obstacle searching range and improved the search efficiency but also greatly improved the dynamic obstacle detection accuracy.
2. A high-precision obstacle detection method in a drivable area without training was proposed. The proposed method was based on the U–V disparity image and the prior information of objects in the drivable area. First, the drivable area was segmented using the U–V disparity image. Then, the partial obstacle was detected based on the properties of the intersection of obstacle and road. Finally, a region growing strategy was applied to obtain the complete obstacle.
3. The proposed method was implemented and tested on the Ubuntu system with a large amount of real data. The experimental results showed that the proposed method can achieve high detection precision and low missed detection rate.

The rest of this manuscript is organized as follows. “Related works” briefly reviews the related literature. We presented our moving obstacle detection method in detail in “Proposed method”. The experimental results on KITTI datasets are demonstrated in “Experiments”. Conclusions and future work are stated in “Conclusions and future work”.

## Related works

Dynamic object detection methods can roughly be classified into four categories: the geometric constrained method^1–6^, the motion compensation method^7–17^, the grid-based method^18,20^, and the deep learning method^21–26^. The methods based on geometric constraints attempt to estimate the background model through the geometric constraint of static points in the scene. The commonly used geometric constraints include affine transformation^2^, homography matrix^3–5^, fundamental matrix^6^, and hybrid geometric model^7^. The basic steps of this category of methods are as follows. First, the matching feature points of two consecutive frames are calculated. Subsequently, the Random Sample Consensus (RANSAC) algorithm is used to sort out inliers that meet a certain constraint and outliers on the dynamic object and estimate the parameters of the model based on the inliers. Finally, the background model is updated with the estimated parameters. However, RANSAC usually fails if moving objects constitute the main part of the image. Moreover, only one constraint cannot be suitable for all scenarios because they fail in their own degenerate case^7^, and these monocular-based methods lack a scale.

The methods based on motion compensation are used to calculate the motion vector generated by the camera ego-motion to realize compensation to transform dynamic background problems into static background problems. These methods are further divided into sparse feature-based methods^8,9,11,12^ and dense optical flow-based methods^1,13–18^. For the former, Lin et al.^8^ used depth information and visual odometry to detect moving objects and designed an adaptive thresholding method to judge the movement state of the target. Lenz et al.^9^ proposed a method for moving object detection based on sparse scene flow. However, sparse feature-based methods fail when few features are detected on the moving objects, such as a scene with insufficient texture features. For the latter, dense optical flow-based methods assume that the optical flow generated by the camera ego-motion (EOF) is mixed with the ROF of the moving object to form the mixed global optical flow (GOF). To obtain the ROF, the GOF can be easily estimated from the existing optical flow algorithms. Subsequently, the camera ego-motion parameters can be calculated with the aid of visual odometry, and then the EOF can be estimated. Based on the aforementioned information, moving objects are detected according to the ROF obtained using the difference between the GOF and EOF^13^. However, the estimation uncertainty, for example, camera motion and disparity, leads to many false positives if ignored. A first-order error propagation model that considers all the estimation errors during the process is used to reduce the influence of the uncertainty, and the moving object is then separated out via the thresholding of Manhattan distance about the uncertainty^14,16^. In 2017, Zhou et al.^1^ built an energy function with three constraints: object color consistency, disparity consistency, and optical flow boundary to improve moving object segmentation. However, the involved global disparity and optical flow computations are time-consuming. To enhance the detection speed, Derome et al.^15^ proposed a very fast OF method^22^ based on LK (Lucas-Kanade) and adopted real-time strategy for the two parts. Chen et al.^17^ proposed a real-time, pixel-level moving object detection method based on stereo cameras. Feature point matching based on RANSAC between consecutive stereo pairs was used instead of dense optical flow calculation.

The methods based on grids take advantage of the high memory efficiency of the occupied grid, distinguish grid cells of the dynamic environment as free or occupied, and then segment and track these cells to provide an object-level detection result. Nguyen et al.^18^ used the binocular information to build a 2D occupancy grid map and then applied a hierarchical segmentation method based on the distance between cells to cluster grid cells into object segments. Li et al.^20^ presented a stereo-vision-based method to build an improved dynamic occupancy grid map. First, the motion estimation for the moving object was obtained by circular feature tracking between two subsequent stereo image pairs. Next, they used the information from the U-disparity map to achieve moving object segmentation in a disparity map. Finally, they estimated the pitch angle between the stereoscopic system and the ground plane to improve the quality of the occupancy grid map.

The core idea of the moving object detection method based on deep learning is to use training data to train network model parameters and hence achieve end-to-end moving object detection. Heo et al.^22^ proposed a network framework for moving object detection based on the deep learning method. The network framework was composed of two networks: A-net and M-net. A-net was used to detect moving objects, such as pedestrians, vehicles, and so forth. The main function of M-net was to detect the movement of objects by comparing the differences between the original image and the background image. Therefore, M-net needed to input not only the image sequence but also the background image generated from the background model. Finally, it combined the appearance information of the object detected by A-net and the motion information detected by M-net to get the moving object in the scene. Li et al.^23^ proposed a method based on the stereo vision to detect and track moving objects. This method combined 2D detection, discrete viewpoint classification, and semantic reasoning to obtain rough 3D object estimation. At the same time, combined with the time sparsity feature, the 3D object pose and velocity were obtained. Siam et al.^24^ proposed a multi-task learning system for moving object detection of intelligent vehicles. The system extracted the appearance features of the RGB image and the motion features of the optical flow image through the VGG network. It then fused the two high-dimensional features and finally realized the moving object detection. Zhe et al.^25^ presented a system that can predict each object’ 3D motions from unlabelled stereo videos. The core idea of the system is formulating a learning objective which works with the limited amount of supervision and factoring the scene representation into independently moving objects. Zhang et al.^26^ utilized scene flow to detect moving objects. By comparing the magnitude of scene flow with a preset threshold, the moving object can be found. Sundaram et al.^27^ combined semantic segmentation and motion cues to estimate the number of moving objects, their motion parameters and perform segmentation. A statistical inference theory is applied to assess the similarities of similar motions in their work. Mohamed et al.^28^ proposed MODETR, a Moving Object Detection Transformer network, comprised of multi-stream transformer encoders for both spatial and motion modalities, and an object transformer decoder that produced the moving objects bounding boxes using set predictions. Hazem et al.^29^ designed a CNN based moving object detection system by utilizing vehicle motion. Their system achieved an absolute improvement of 5.6% in mIoU over the baseline architecture. Prarthana et al.^30^ reported the first systematic exploration and assessment of incorporating self-supervision into motion forecasting for autonomous driving. Min et al.^31^ proposed an object-level motion segmentation method for multiple moving objects combining geometric methods and deep learning. First, a depth and pose estimation framework and an instance segmentation network was used to estimate the depth, pose, and instance segmentation, then an instance re-projection residual was applied to estimate the moving states.

## Proposed method

**Figure 1:**
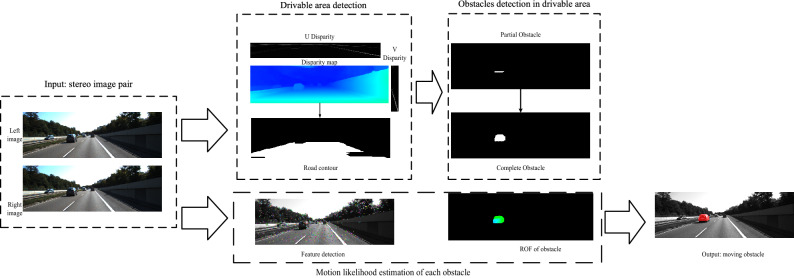
Overall flow chart of the algorithm (the input images are from KITTI dataset^36^).

In this section, the details of the proposed method for moving obstacle detection using a vehicle-mounted binocular camera were discussed. As shown in Fig. 1, the input of the system was binocular video images. Since complex scenarios with many details (such as trees, buildings, and so on) in the image are often distributed on both sides of the road, we first detected the drivable area in front of the unmanned vehicle to eliminate the interference of complex scenes. We then detected the obstacles in the drivable area. Finally, the motion likelihood of each obstacle was calculated to determine the motion state.

### Drivable area detection

**Figure 2:**
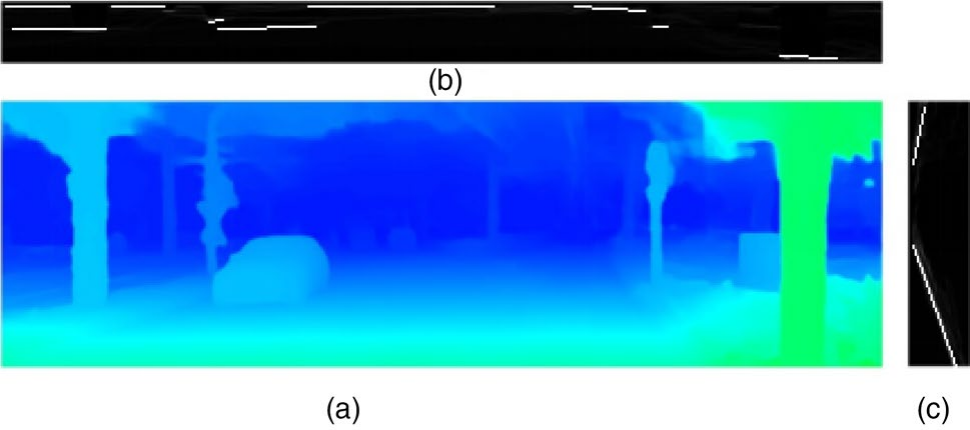
An example of a U–V disparity image. (**a**) Disparity image. (**b**) U-disparity image. (**c**) V disparity image.

In this study, U–V disparity images were applied to detect the drivable area of unmanned vehicles. The concepts of U–V disparity images were first proposed in Ref.^32^, which could be used to detect the drivable area and obstacles without prior knowledge. U–V disparity images are composed of V disparity image and U-disparity image, which can both be computed from the disparity map. V disparity image is built by projecting the disparity map horizontally, where all the road surface disparity points are projected on a line. U-disparity image, on the contrary, is produced by projecting the disparity image vertically, which provides a top-view projection of the scene and retains more obstacle information, such as the width of obstacles, the relative position relationship between obstacles, and so forth. Figure 2 illustrates an example of the disparity map and its corresponding U-disparity image and V disparity image. In this study, the drivable area detection from U–V disparity images included four parts.

#### Crude obstacle removal

**Figure 3:**
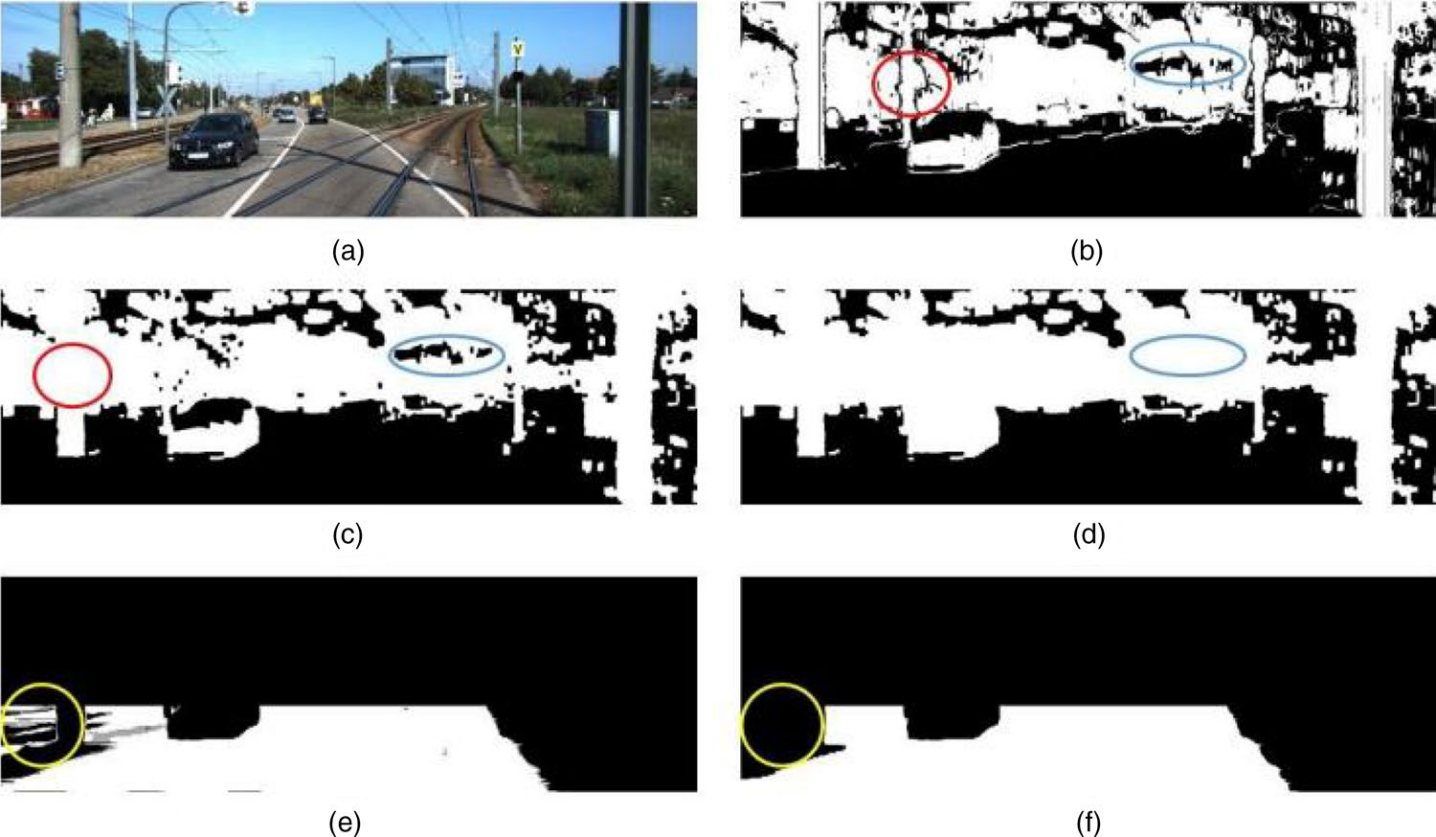
(**a**) Original image. (**b**) Initial binarized obstacle image. (**c**) Morphological close operation (narrow gaps lie in the red circle). (**d**) Eliminate isolated small areas (isolated small areas lie in the blue circle). (**e**) Road initial contour (noise lies in the yellow circle). (**f**) Road contour purification.

The purpose of this step was to remove crude obstacles in the scene, such as buildings, trees, vehicles parked on the side of the road, and so forth. Since the disparity values of obstacles perpendicular to the road surface in the disparity map were similar, the disparity values belonging to the same obstacle formed a peak in a certain column in the U-disparity image. Therefore, a thresholding operation was designed to remove these obstacles. As shown in Eq. (1), if the value of $U\left(u,d\right)$ was larger than $\tau$, the pixel ($u,v$) in the disparity map was regarded as an obstacle pixel. Otherwise, it was a pixel belonging to the road, where $U$, $D$ denote the U-disparity image and disparity image, respectively, and $\tau$ is a threshold. After this step, a binarized image $Obstaclemask$, denoted by that distinguished the roads from the obstacles, was obtained, as shown in Fig. 3b. In Fig. 3b, the white area represents the obstacles, and the black area is the crude passable area. In this study, $\tau$ was set as 10–15 pixels empirically.

1 $$Obstaclemask\left( {u,v} \right) = \left\{ \begin{gathered} \begin{array}{*{20}c} 1 & {if\;U\left( {u,d} \right) > \tau ,\;d\; = \;D\left( {u,v} \right)} \\ \end{array} \hfill \\ \begin{array}{*{20}c} 0 & {otherwise} \\ \end{array} \hfill \\ \end{gathered} \right.$$

#### Noise filtering

Due to the inevitable error of disparity estimation, especially in an area with discontinuous disparity, the obstacles may be misjudged as the roads, as marked in Fig. 3b, and Fig. 3a is the corresponding original image. Misjudged regions are of two types. One is the narrow gap marked in red circle in Fig. 3b, which usually exists at the junction of two obstacles with discontinuous disparity. The other is the isolated small area, which is often caused by the invalid disparity value, as shown in blue ellipse in Fig. 3b. For the narrow gaps, a morphological close operation was performed to link small areas between potential obstacles in this study, as shown in Fig. 3c. The isolated small areas could be eliminated by detecting the contour and carrying out an area constraint operation, as shown in Fig. 3d. After this step, the crude drivable area could be refined.

#### Road detection

For an unmanned vehicle, the drivable area refers to the road area in front of it. An efficient nonparametric road surface detection algorithm^33^ was used to obtain the road area. The core idea of this method was that it exploited the depth cue to find most of the the initial road profile points according to the fact that the points with the maximum value for each row in the V disparity image were more likely to be the projecting points of the lateral road lines after removing the obstacles. In this study, we only provided the road detection result, as shown in Fig. 3e. More details about this method can be found in^28^. As shown in Fig. 3e, some obstacles on both sides of the road, marked in a yellow circle, were mistakenly detected as roads. For this case, an area threshold operator was carried out to remove these mistakenly detected roads since the road occupied a main part of the image, and the result is shown in Fig. 3f.

### Obstacles detection in drivable area

When the road is detected, the search range of the obstacles in the image is limited. The existing obstacle detection methods^34,35^ mainly concentrated on using U-disparity image information after removing the road area. However, it was difficult to separate the obstacles from the road completely only depending on the U-disparity image. In this study, a two-step obstacle detection method was proposed. The whole process of the proposed algorithm included two parts: partial obstacle detection and complete obstacle detection.

#### Partial obstacle detection

**Figure 4:**
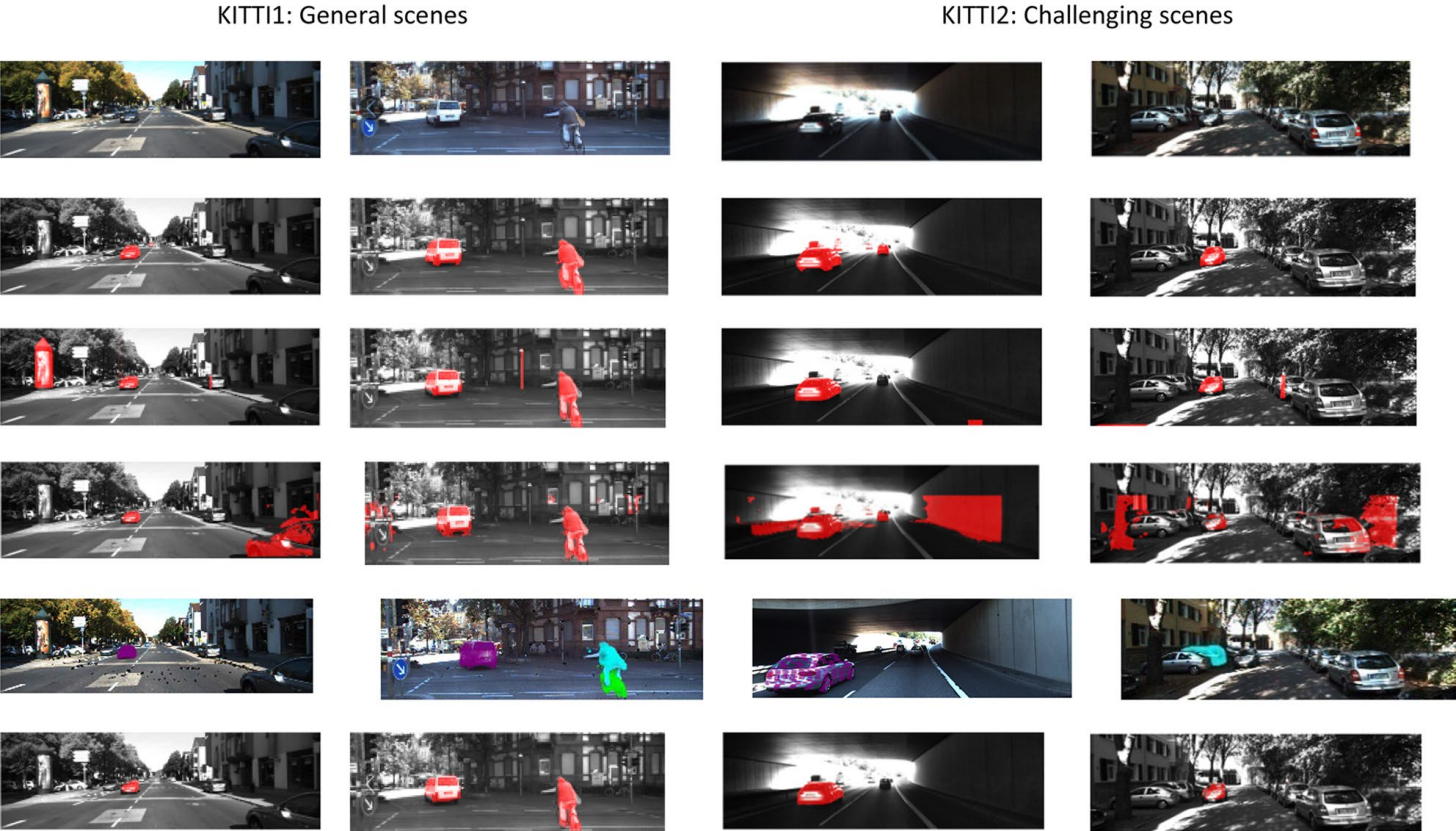
Results of moving object detection on general and challenging scenes (KITTI dataset). The first line represents the original image; Line 2 is the ground truth; Lines 3 to 6 are the results using Lin’s, Zhou’s, Zhang’s, and our methods, respectively. The first two columns are examples of general scenes and the last two columns are examples of challenging scenes.

The obstacles occupy a certain part of the road, and hence the road is partially occluded. Therefore, a gap exists where the obstacles are in the image, as shown in Fig. 4. Based on this attribute, the problem of partial obstacle detection is transformed into the problem of gap location in the road area.

However, some gaps are generated by noise, which are not the real obstacles. A filtering criterion was designed to filter out these gaps. Given the camera parameters, we derived the mathematical relationship between the obstacle size and its disparity, as shown in Eq. (2).

2 $$\left\{ {\begin{array}{*{20}c} {X_{\min } = (u_{\min } - C_{u} ) \times {Z \mathord{\left/ {\vphantom {Z f}} \right. \kern-0pt} f}} \\ {X_{\max } = (u_{\max } - C_{u} ) \times {Z \mathord{\left/ {\vphantom {Z f}} \right. \kern-0pt} f}} \\ \end{array} } \right. \Rightarrow W = \left( {X_{\max } - X_{\min } } \right) = (u_{\max } - u_{\min } ) \times {b \mathord{\left/ {\vphantom {b {\overline{d}}}} \right. \kern-0pt} {\overline{d}}}$$

where $W$ denotes the width of the obstacle, $Z$ is the distance, and $f$ is the focal length of the camera, ${C}_{u}$ denotes the projection of the x coordinate of the camera optical center in the image plane, $b$ is the baseline of the camera, and $\overline{d}$ is the average disparity value of the current row of the gap. Usually, the obstacles on the road are vehicles and pedestrians, and their widths have a priori value. Given the value of $W$, $b$, and $\overline{d}$, the width of the obstacle in the $X$ direction of the image can be calculated using Eq. (2). Therefore, the gaps generated by noise can be filtered out by comparing the calculated width of the obstacle and the width of the obstacle in the image.

A contour defect detection algorithm was used to obtain the region of the obstacle on the road based on disparity similarity and the relationship between the width of objects and disparity after filtering out the gaps generated by noise. The region was one part of the whole obstacle, named partial obstacle in this study.

#### Complete obstacle detection

A region growing algorithm was designed to obtain a complete obstacle from the detected partial obstacle. Usually, a region growing algorithm contains three essential factors: seed points, growth criterion, and stop condition. The center point of the partial obstacle is set as a seed point. In this way, the influence of partial obstacle error on the complete obstacle detection can be avoided as much as possible. The criterion for regional growth is disparity similarity. When the disparity between the seed point and its neighborhood is less than a threshold, the neighborhood pixel is marked as a new seed point. The following stop constraints were designed to prevent excessive and false growth: (1) the growth range is the non-road area in the image, and (2) the width of each obstacle is close to their prior value.

### Motion likelihood estimation of each obstacle

After detecting the obstacles in the passable area, the next step is to judge the motion state of each obstacle. Since the camera is moving, a direct method is to compensate the motion vector generated by the camera ego-motion. In this way, the problem of obstacle motion state estimation in the moving background is transformed into the estimation of the obstacle motion state in the static background. The motion state estimation of an obstacle includes three parts: camera ego-motion estimation, residual flow estimation, and motion likelihood estimation.

#### Camera ego-motion estimation

Let {$R,T$} denote the camera ego-motion parameters, from time $t - 1$ to $t$, $x_{t}^{i} \in R^{2}$ is the feature point in the frame $t$, and $P_{t - 1}^{i} \in R^{3}$ is the 3D point of $x_{t}^{i}$ in the camera coordinate system at the time $t - 1$. Based on the principle of binocular geometry, $\left\{ {R,T} \right\}$ can be estimated by minimizing the sum of the reprojection errors using nonlinear minimization approaches, as shown in Eq. (3), where $\rho$ is the Huber cost function, $\Sigma$ is the covariance matrix, and $\pi$ is the projection function of the pinhole camera model. More detail can be found in^7^.

3 $$\left\{ {R,T} \right\} = \mathop {\arg \min }\limits_{R,T} \sum\limits_{i} {\left( {\rho \left( {\left\| {x_{t}^{i} - \pi (RP_{t - 1}^{i} + T)} \right\|_{\sum }^{2} } \right)} \right)}$$

#### Residual flow estimation

Given a static pixel point $p_{t - 1} = \left( {u_{t - 1} ,v_{t - 1} ,1} \right)^{T}$ in the frame $t - 1$, its corresponding point $p_{t} = \left( {u_{t} ,v_{t} ,1} \right)^{T}$ in the frame $t$ can be predicted using Eq. (4), where $K$ is the camera intrinsic parameter matrix, and $Z_{t - 1}$ is the depth at time $t - 1$. Then, the optical flow caused only by camera motion can be calculated using Eq. (5). Finally, the residual flow caused only by the movement of the obstacle itself can be expressed using Eq. (6), where $m = \left( {m_{u} ,m_{v} } \right)$ is the global optical flow and $q$ is the residual flow.

4 $${\text{p}}_{t} = KRK^{ - 1} p_{t - 1} + \frac{KT}{{Z_{t - 1} }}$$

5 $$g = (g_{u} ,g_{v} )^{T} = (u_{t} - u_{t - 1} ,v_{t} - v_{t - 1} )^{T}$$

6 $$q = g - m = (g_{u} - m_{u} ,g_{v} - m_{v} )^{T}$$

#### Motion likelihood estimation

Theoretically, if a point is stationary, its residual flow is 0; otherwise, it is larger than 0. However, errors are always found in the residual flow due to the inevitable errors in optical flow and the matching calculation of two images involved in the whole calculation process of residual flow estimation. Therefore, if we simply use a fixed threshold to judge the motion state of a pixel, the result is unsatisfactory. To solve the above problems, Ref.^14^ proposed a method considering the estimation error of each stage in the whole calculation process. A first-order error forward propagation model was used to transfer the uncertainty from the sensor to the final result. For each obstacle, the Manhattan distance of the residual flow was used to measure the motion possibility of each pixel to judge its motion statement, and then these moving pixels were clustered. When the number of pixels was greater than a threshold, the moving state of the corresponding obstacle was regarded as moving.

## Experiments

This section describes the experiments carried out to evaluate the performance of the proposed algorithm.

### Experimental preparation

#### Testing data

In this study, the same testing data in Ref.^17^ and our self-acquired campus scene data, named CUIT-CS, were used for the experiment. In Ref.^17^, 304 dynamic scenes, including different illumination, different road types, and different kinds of moving targets from the KITTI dataset, were selected as testing samples. Each scene was described by two pairs of binocular images acquired at consecutive times with a resolution of 1242 × 375 pixels. We divided the data into two subsets: KITTI1 and KITTI2. In KITTI1, the scene was simple, and only the vehicles were moving objects. In KITTI2, the moving object and scene structure were more complex. A previous study^36^ described the sensors used and date information at great length. In ourself-acquired campus scene dataset, stereo images at 14 different scenes in the Chengdu University of Information Technology (CUIT) were collected; each scene had 200 pairs of consecutive images. The ground truth of the moving obstacles both from the KITTI benchmark and our CUIT-CS dataset were marked in pixels manually.

All experiments in this study were completed on a desktop computer whose processor was Intel Core i5-8300H CPU 2.3 GHz with 16-GB RAM and GTX 1060. The experimental environment was Ubuntu 16.04 with OpenCV 3.4.7.

#### Parameters setting

In this study, the threshold $\tau$ in formula (1) was set to 15 pixels. The width of the vehicle was assumed from 1.5 to 3 m, and the width of people was assumed from 0.2 to 0.7 m. The detection distance were limited from 0 to 25.3 m for KITTI dataset, and from 0 to 10 m for CUIT dataset.

#### Evaluation criteria

The precision $P$, recall $R$, and F-measure $F$ were used to measure the performance of the algorithms to quantitatively analyze the performance of the proposed method. These evaluating indicators were defined as follows.

7 $$P = \frac{tp}{{tp + fp}},R = \frac{tp}{{tp + fn}},F{ = }\frac{2R \times P}{{R + P}}$$

In Eq. (7), $tp$ represents the number of moving obstacles detected as moving obstacles, $fp$ represents the number of static obstacles detected as moving obstacles, and $fn$ represents the number of moving obstacles that have not been detected. Equation (8) is used to measure whether an object is moving, where $B_{g}$ and $B_{d}$ are the ground truths of moving obstacles and detected pixels of moving obstacles, respectively. In this study, when the *score* was greater than or equal to 0.5, the obstacle was considered to be moving.

8 $$score = \frac{{B_{g} \cap B_{d} }}{{B_{g} \cup B_{d} }}$$

### Experimental results and analysis

#### Quantitative evaluation

In this study, the core principle of moving obstacle detection was based on the ROF. Zhou^7^, Lin^8^ and Zhang^26^’s work were chosen to be comparison algorithms. Zhou and Lin’s work are the typical studies of dense and sparse ROF-based moving obstacle detection; Zhang’s work is scene flow based. In this section, we quantitatively analyzed the performance of the four algorithms.

Table 1 shows the performance of each method for moving obstacle detection on KITTI1, KITTI2, and CUIT-CS datasets. Column 1 in Table 1 shows the four methods to be used for comparative experiments. Column 2 shows the evaluation index. Columns 3–5 are the detection results on KITTI1, KITTI2, and CUIT-CS datasets, respectively. Column 6 shows the average detection results on all the datasets. As shown in Table 1, Lin’s method has the lowest precision, and the recall is only 0.645. Zhou’s method has higher recall, close to 0.8, however the precision is only 0.572. Zhang’s method has the highest precision, but the recall is 0.511, the lowest compared to other algorithms, which means many moving objects could be missed. The precision of our method is 0.734, which is lower than Zhang’s method; However, the recall of our method is 0.825, which is much higher than Zhang’s method, and is also the highest in the comparison algorithms. F is the comprehensive evaluation index of precision and recall rate. Our method has the best F, reaching to 0.827, which shows our method can achieve high detection precision and low missed detection rate.

**Table 1:** Comparison of the experimental results using two different algorithms.

Method	Evaluation	KITTI1	KITTI2	CUIT-CS	Average
Zhou’s	P	0.697	0.610	0.41	0.572
	R	0.721	0.767	**0.91**	0.799
	F	0.708	0.680	0.57	0.653
Lin’s	P	0.227	0.452	0.45	0.376
	R	0.492	**0.883**	0.56	0.645
	F	0.312	0.598	0.50	0.47
Zhang’s	P	**0.892**	**0.855**	**0.937**	**0.895**
	R	0.588	0.613	0.333	0.511
	F	0.709	0.714	0.491	0.638
Ours	P	0.825	0.783	0.894	0.734
	R	**0.818**	0.853	0.805	**0.825**
	F	**0.821**	**0.816**	**0.844**	**0.827**

Significant values are in bold.

#### Qualitative analysis

**Figure 5:**
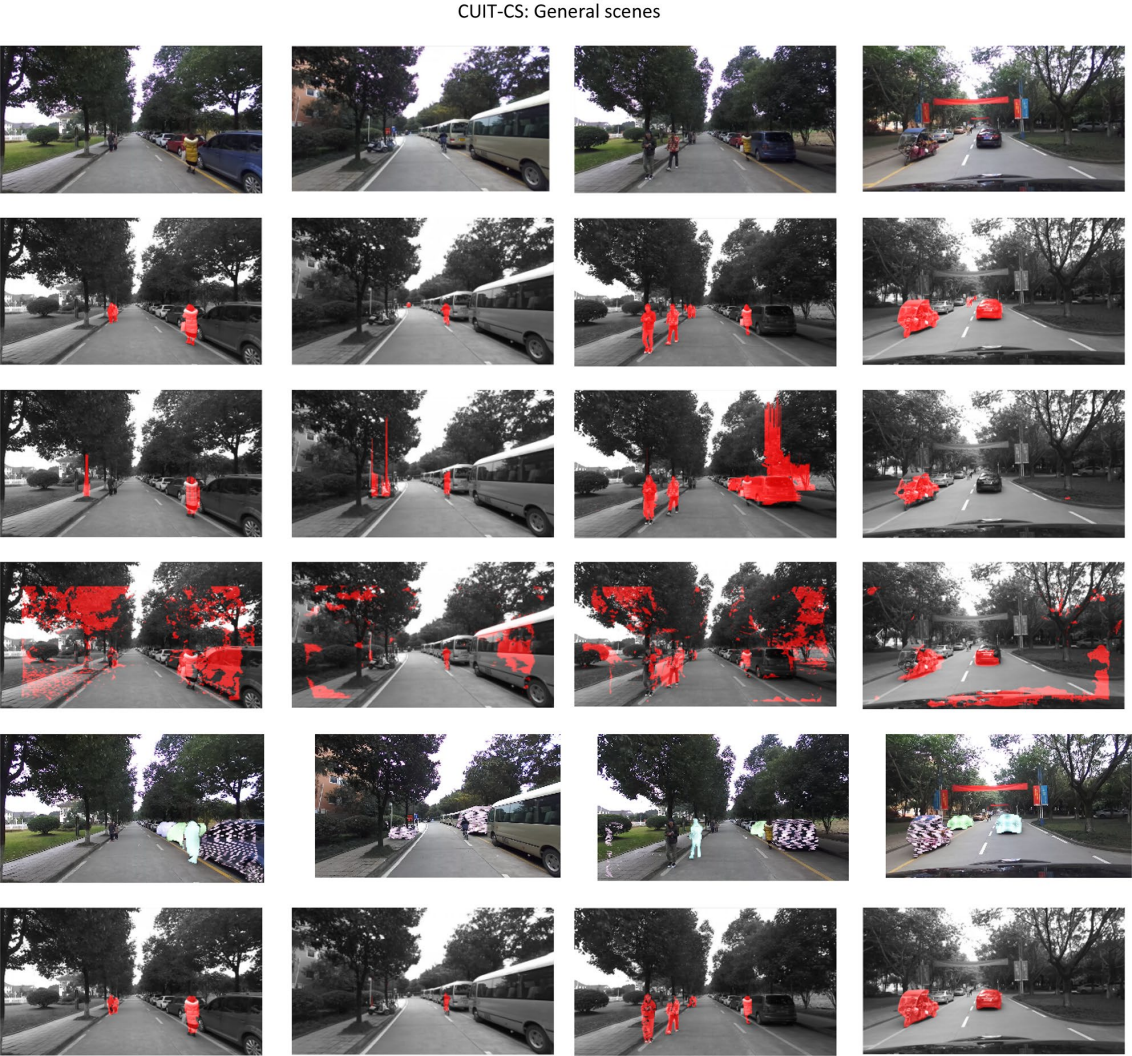
Results of moving object detection on general scenes (our CUIT-SD dataset). The first line represents the original image; Line 2 is the ground truth; Lines 3 to 5 are the results using Lin’s, Zhou’s, Zhang’s, and our methods, respectively.

Figures 4, 5 shows the results of moving object detection on KITTI dataset and our CUIT-CS dataset. In Figs. 4 and 5, the first line represents the original image; the second line shows the ground truth; the third to sixth lines are the results using Lin’s, Zhou’s, Zhang’s, and our methods, respectively. In Fig. 4, the first two columns are the results on general scenes from KITTI1, and the last two columns are the results on challenging scenes from KITTI2. From the first two columns of Fig. 4, it can be seen that all the traffic participants, namely vehicles, are moving, and most areas of the scene are uniformly illuminated. The third column of Fig. 4 shows a tunnel scene under low-light conditions containing vehicles and walls; the illumination at the tunnel entrance and in the tunnel varies significantly. The fourth column of Fig. 5 shows the road environment of urban residential areas. A lot of trees are shown on both sides of the road, forming a large area of shadow on the road, and many vehicles are parked on both sides.

As can be seen from the third line of Fig. 4, Lin's method can detect most moving objects, and there are only a few false detections. From the line 4 of Fig. 4, although Zhou's method can detect all moving objects, it also misjudges many static targets, like walls and trees, as moving objects. Zhang's method has missed detection and false detection for challenging scenes (See line 5, column 4). The sixth line of Fig. 4 shows the results of our method. It can be seen that our method can effectively detect moving objects in both general and challenging scenes. In the four scenes listed, only the vehicles running at the tunnel exit are not detected in the tunnel scene.

Figure 5 shows the results of comparative experiments on our CUIT-SD dataset. The experimental results are the same as those on the KITTI data set. Lin's and Zhang’s method have a small amount of false detection and missed detection; Zhou's method had almost no missed detection, but the false detection is very serious; The algorithm proposed in this paper can detect all moving objects except small moving objects in the distance, and there are few false detections.

#### Operation time

Essentially, our method was a dense ROF-based method. Therefore, in this section, only the ROF-based method is chosen as the comparison algorithm. Zhou’s method is a representative method based on the ROF. In Zhou’s method, the ROF of the whole image is computed, and then moving objects are extracted from the whole ROF. The difference between Zhou’s method and our method was that we calculated the ROF only in the area where the obstacle was in the drivable area. Therefore, in our method, although the process of obstacle detection was added, the searching range of moving objects was reduced. A total of 100 scenes were randomly selected from the dataset as the testing samples to test the computational efficiency of our algorithm. The experimental result is shown in Table 2. Compared with Zhou's method, although our method added the obstacle extraction from the drivable area of unmanned vehicles before the motion likelihood computation, it was 3.346 s faster than Zhou's method. The reason why our algorithm was faster was that the searching range of moving obstacles in the image was greatly narrowed using U–V disparity images.

**Table 2:** Comparison of the time consumption of the two algorithms.

Method	Time (ms)
Ours	61
Zhou’s	3407

## Conclusion and future work

In this study, a moving object detection method based on two cameras was proposed, aiming at the problem of moving obstacle detection with high precision in complex scenarios for unmanned vehicles. The new point of the proposed method was that not only the searching range of dynamic obstacles could be reduced, but also the pseudo moving obstacles outside the current road could be eliminated by fusing drivable area detection and dense residual flow estimation. In order to improve the detection accuracy, the algorithm designed in this paper used some predefined parameters and thresholds. Once those parameters and thresholds are fixed, the algorithm may be not robust to different scenarios. So, in order to improve the adaptability of the algorithm to different scenarios, we plan to integrate instance segmentation and sparse residual flow into our system in the future.

## Data Availability

The datasets used during the current study available from the corresponding author.

## References

[1] Zhou, D., Wang, L., Cai, X. & Liu, Y. Detection of moving targets with a moving camera. in *Proceedings of the ROBIO**, *Guilin, Guangxi, China, Dec 19–23, 2009. 10.1109/ROBIO.2009.5420591 (2009).

[2] Liu, F. & Gleicher, M. Learning color and locality cues for moving object detection and segmentation. in *Proceedings of the CVPR,* Miami, FL, USA*,* June 20–25*,* 2009. 10.1109/cvprw.2009.5206678 (2009).

[3] Minematsu T, Uchiyama H, Shimada A, Nagahara H, Taniguchi R (2017). Adaptive background model registration for moving cameras. Pattern Recognit. Lett..

[4] Jin, Y., Tao, L. & Xu, G. Background modeling from a free-moving camera by multi-layer homography algorithm. in *Proceedings of the ICIP, *San Diego, USA, Oct12–15, 2008. 10.1109/ICIP.2008.4712069 (2008).

[5] Jung Y (2017). An efficient fundamental matrix estimation for moving object detection. Int. J. Comput. Inf. Eng..

[6] Zamalieva, D., Yilmaz, A. & Davis, J.W. A multi-transformational model for background subtraction with moving cameras. in *Proceedings of the ECCV,* Zurich, Switzerland, Sep 5–12, 2014 (2014).

[7] Zhou D, Fremont V, Quost B, Dai Y (2017). Computing, "moving object detection and segmentation in urban environments from a moving platform". Image Vis. Comput..

[8] Lin, S.F., & Huang, S.H. Moving object detection from a moving stereo camera via depth information and visual odometry. in *Proceedings of the IEEE ICASI,* Chiba, Tokyo, Japan, Apr13–17, 2018 (2018).

[9] Lenz, P., Ziegler, J., Geiger, A. & Roser, M. Sparse scene flow segmentation for moving object detection in urban environments. in *Proceedings of the IV*, Baden-Baden, Germany, June 05–09, 2011. 10.1109/IVS.2011.5940558 (2011).

[10] Kazunori (2017). Moving object detection using a stereo camera mounted on a moving platform. SICE J. Control Meas. Syst. Integr..

[11] Toda, T., Masuyama, G. & Umeda, K. Detecting moving objects using optical flow with a moving stereo camera. in *Proceedings of the MOBIQUITOUS 2016: Computing Networking and Services*, Hiroshima, Japan, Nov 28–Dec 1 (2016).

[12] Yoo J, Lee G (2019). Moving object detection using an object motion reflection model of motion vectors. Symmetry.

[13] Zhou, D., Frémont, V. & Quost, B. Moving objects detection and credal boosting based recognition in urban environments. in *Proceedings of the CIS & RAM,* Manila, Philippines, Nov 12–15, 2013. 10.1109/ICCIS.2013.6751573 (2013).

[14] Zhou, D., Frémont, V., Quost, B. & Wang, B. On modeling ego-motion uncertainty for moving object detection from a mobile platform. in *Proceedings of the IV*, Dearborn, USA. 1332–1338 (2014).

[15] Derome M, Plyer A, Sanfourche M, Besnerais G (2015). Moving object detection in real-time using stereo from a mobile platform. Unmanned Syst..

[16] Derome, M., Plyer, A., Sanfourche, M. & Le Besnerais, G. Real-time mobile object detection using stereo. in *Proceedings of the ICARCV,* Singapore, Dec10–12, 2014. 1021–1026 (2014).

[17] Chen L, Fan L, Xie G, Huang K (2017). Moving-object detection from consecutive stereo pairs using slanted plane smoothing. IEEE Trans. Intell. Transp. Syst..

[18] Nguyen T-N, Michaelis B, Al-Hamadi A, Tornow M (2011). Stereo-camera-based urban environment perception using occupancy grid and object tracking. IEEE Trans. Intell. Transp. Syst..

[19] Shen, S., Kerofsky, L. & Yogamani, S. *Optical Flow for Autonomous Driving: Applications, Challenges and Improvements*. *arXiv preprint* arXiv:2301.04422 (2023).

[20] Li Y, Ruichek Y (2014). Occupancy grid mapping in urban environments from a moving on-board stereo-vision system. Sensors.

[21] Plyer A, Le Besnerais G (2016). Massively parallel Lucas Kanade optical flow for real-time video processing applications. J. Real-Time Image Process..

[22] Heo, B., Yun, K. & Choi, J.Y. Appearance and motion based deep learning architecture for moving object detection in moving camera. in *Proceedings of the ICIP,* Beijing*,* China*,* Sep 17–20, 2017. 1827–1831 (2017).

[23] Li, P. & Qin, T. Stereo vision-based semantic 3D object and ego-motion tracking for autonomous driving. in *Proceedings of the ECCV,* Munich, Germany, Sep 8–14, 2018. 765−781 (2018).

[24] Siam, M., Mahgoub, H., Zahran, M., Yogamani, S., Jagersand, M. & El-Sallab, A. Modnet: Motion and appearance based moving object detection network for autonomous driving. in *Proceedings of the ITSC,* Maui, Hawaii, USA, Nov 04–07, 2018. 2859–2864 (2018).

[25] Cao, Z. *et al*. Learning independent object motion from unlabelled stereoscopic videos. in *Proceedings of the IEEE/CVF Conference on Computer Vision and Pattern Recognition*. 5594–5603 (2019).

[26] Zhang, J. *et al*. Robust ego and object 6-DoF motion estimation and tracking. in *2020 IEEE/RSJ International Conference on Intelligent Robots and Systems* (*IROS*) (IEEE, 2020).

[27] Muthu, S. *et al*. Motion segmentation of rgb-d sequences: Combining semantic and motion information using statistical inference. in *IEEE Transactions on Image Processing*. Vol. 29. 5557–5570 (2020). 10.1109/TIP.2020.2984893 32275594

[28] Mohamed, E., & El-Sallab, A. *Modetr: Moving Object Detection with Transformers*. *arXiv preprint* arXiv:2106.11422 (2021).

[29] Rashed, H., El Sallab, A. & Yogamani, S. VM-MODNet: Vehicle motion aware moving object detection for autonomous driving. in *2021 IEEE International Intelligent Transportation Systems Conference* (*ITSC*). 1962–1967 (IEEE, 2021).

[30] Bhattacharyya, P, Huang, C, & Czarnecki, K. Ssl-lanes: Self-supervised learning for motion forecasting in autonomous driving. in *Conference on Robot Learning*. 1793–1805 (PMLR, 2023).

[31] Yue M (2022). Vehicle motion segmentation via combining neural networks and geometric methods. Robot. Auton. Syst..

[32] Labayrade, R., Aubert, D. & Tarel, J.-P. Real time obstacle detection in stereovision on non flat road geometry through "V-disparity" representation. in *Proceedings of IV,* Versailles, France, Jun 17–21, 2002. 646–651 (2002).

[33] Wu M, Lam S (2014). Nonparametric technique based high-speed road surface detection. IEEE Trans. Intell. Transp. Syst..

[34] Wang, B. Florez, V. & Frémont, V. Multiple obstacle detection and tracking using stereo vision: Application and analysis. in *Proceedings of the ICARCV,* Singapore, Dec 10–12, 2014. 1074–1079 (2014).

[35] Dekkiche, D., Vincke, B. & Mérigot, A. Vehicles detection in stereo vision based on disparity map segmentation and objects classification. in *Proceedings of the ISVC,* Las Vegas, Nevada, USA, Dec. 14–16, 2015. 762–773 (2015).

[36] Geiger, A., Lenz, P. & Urtasun, R. Are we ready for autonomous driving? The KITTI vision benchmark suite. in *Proceedings of the CVPR,* Providence, RI, USA, June 16–21, 2012. 10.1109/CVPR.2012.6248074 (2012).

